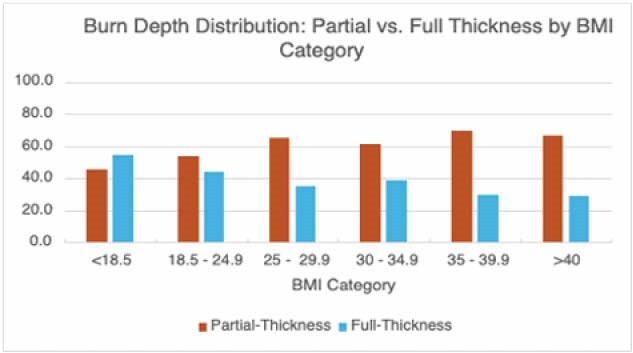# 882 Exploring Burn Depth Patterns: Higher Body Mass Index Is Protective for Full-Thickness Burns

**DOI:** 10.1093/jbcr/iraf019.413

**Published:** 2025-04-01

**Authors:** Rebecca Hohsfield, Hilary Liu, José Arellano, Christopher Fedor, Mare Kaulakis, Alexis Henderson, Garth Elias, Alain Corcos, Jenny Ziembicki, Francesco Egro

**Affiliations:** University of Pittsburgh Medical Center; University of Pittsburgh Medical Center; University of Pittsburgh Medical Center; University of Pittsburgh School of Medicine; University of Pittsburgh School of Medicine; University of Pittsburgh School of Medicine; University of Pittsburgh Medical Center; University of Pittsburgh Medical Center; University of Pittsburgh Medical Center; University of Pittsburgh Medical Center

## Abstract

**Introduction:**

Burn injuries present complex challenges in clinical management, with burn depth critically influencing treatment and outcomes. Understanding factors that affect burn depth severity is therefore crucial. While existing literature addresses various demographic and physiological factors that affect burn depth severity, such as patient age and skin composition, there is a notable gap in understanding how Body Mass Index (BMI) influences burn depth patterns. This study aims to analyze burn depth patterns across BMI categories to elucidate the role of adipose tissue in mitigating injury severity.

**Methods:**

A retrospective review was conducted on patients who sustained acute lower extremity burns at a single ABA-verified burn center between January 2012 and August 2022. Patients were divided into six BMI groups based on World Health Organization classification; underweight: < 18.5, normal: 18.5-24.9, overweight: 25-29.9, obesity class 1: 30-34.9, class 2: 35-39.9, and class 3: >40kg/m². Burn injuries were classified as partial-thickness versus full-thickness. Statistical analysis was performed via two-tailed chi-square tests to identify whether an association exists between BMI and burn depth.

**Results:**

A total of 409 patients were analyzed (32% female, 68% male; mean age of 47.6±19.2 years). The mean BMI among the cohort was 28.6±6.5 kg/m2, with most patients in the healthy weight (n=124; 30.3%) and overweight (n=129; 31.5%) categories, followed by class 1 obesity (n=81; 19.8%), class 2 obesity (n=40; 9.8%), class 3 obesity (n=24; 5.9%), and underweight (n=11; 2.7%).

Most patients had injuries secondary to flame (n=218; 53.3%), followed by scald (n=123; 30.1%), contact (n=35; 8.6%), chemical (n=14; 3.4%) and electrical burns (n=5; 1.2%). A similar distribution was present when analyzed among individual BMI categories. The range of partial to full-thickness burns widened with increasing BMI, with a lesser proportion of overweight, obese class 1, 2, and 3 patients experiencing full-thickness burns (Figure 1). Chi-squared analysis revealed a significant association when comparing burn thickness in patients with a BMI< 25 kg/m² (underweight, normal weight) to patients with a BMI>25 kg/m² (overweight and above); χ2(1)=4.35; p=0.037.

**Conclusions:**

This study demonstrates a significant association between BMI and burn depth severity, indicating that while higher BMI may confer a protective effect against full-thickness burns, lower BMI may be an important risk factor.

**Applicability of Research to Practice:**

Understanding this relationship may guide healthcare professionals in tailoring treatment strategies, such as expected versus actual fluid resuscitation requirements following burn injury. Conversely, recognizing that lower BMI is associated with a heightened risk of full-thickness burns warrants more careful consideration when applying controlled heat in medical settings, as these patients may be more susceptible to thermal injuries.

**Funding for the Study:**

N/A